# The Early Peritoneal Cavity Immune Response to *Vibrio Anguillarum* Infection and to Inactivated Bacterium in Olive Flounder (*Paralichthys olivaceus*)

**DOI:** 10.3390/microorganisms10112175

**Published:** 2022-11-02

**Authors:** Xueyan Shi, Heng Chi, Yuanyuan Sun, Xiaoqian Tang, Jing Xing, Xiuzhen Sheng, Wenbin Zhan

**Affiliations:** 1 Laboratory of Pathology and Immunology of Aquatic Animals, KLMME, Ocean University of China, 5 Yushan Road, Qingdao 266003, China; 2 Laboratory for Marine Fisheries Science and Food Production Processes, Qingdao National Laboratory for Marine Science and Technology, Qingdao 266071, China; 3 Key Laboratory of Experimental Marine Biology, Institute of Oceanology, Chinese Academy of Sciences, Qingdao 266071, China

**Keywords:** peritoneal cavity cells, cell composition, infection, vaccination, fish

## Abstract

The peritoneal cavity plays an important role in the immune response, and intraperitoneal administration is an ideal vaccination route in fish. However, immune responses in the peritoneal cavity of teleost fish are still not completely characterized. This study characterized the morphology of peritoneal cavity cells (PerC cells) and their composition in flounder (*Paralichthys olivaceus*). Flow cytometric analysis of the resident PerC cells revealed two populations varying in granularity and size. One population, approximately 15.43% ± 1.8%, was smaller with a lower granularity, designated as lymphocytes. The other population of the cells, about 78.17% ± 3.52%, was larger with higher granularity and was designated as myeloid cells. The results of cytochemical staining and transmission electron microscopy indicated that peritoneal cavity in flounder normally contains a resident population of leukocytes dominated by granulocytes, macrophages, dendritic cells, and lymphocytes. The percentages of IgM^+^, CD4^+^, G-CSFR^+^, MHCII^+^, and CD83^+^ leukocytes among PerC cells determined by flow cytometry were 3.13% ± 0.4%, 2.83% ± 0.53%, 21.12% ± 1.44%, 27.11% ± 3.30%, and 19.64% ± 0.31%, respectively. Further, the changes in IgM^+^, CD4^+^, G-CSFR^+^, MHCII^+^, and CD83^+^ leukocytes in flounder after *Vibrio anguillarum* infection and immunization were compared. The composition changed rapidly after the infection or vaccination treatment and included two stages, a non-specific stage dominated by phagocytes and a specific immune stage dominated by lymphocytes. Due to the virulence effectors of bacteria, the infected group exhibited a more intense and complicated PerC cells immune response than that of the immunization group. Following our previous study, this is the first report on the morphology and composition of PerC cells and the early activation of PerC cells in flounder response to *V. anguillarum* infection and vaccination.

## 1. Introduction

Peritoneal cavity cells (PerC cells) play essential roles in innate and adaptive immune responses and are used as a source of leukocytes for cell-mediated immunity studies [1]. The peritoneal cavity defines a confined micro-environment, which is stable under normal conditions. When exposed to infections that cause damaging effects, the recruitment, proliferation, and activation of various immune cells from other immune organs occurs to defend against invasion [2]. In teleosts, the resident peritoneal leukocytes are known to include lymphocytes and myeloid cells (dendritic cells, macrophages, and granulocytes) [3,4,5,6]. Lymphocytes are essential cellular components of the adaptive and innate immune defense. They are also the primary executors of nearly all immune functions of the lymphatic system and soldiers fighting external infections [7]. Dendritic cells (DCs) are the most potent antigen-presenting cells (APCs) of the immune system and possess superior abilities to activate naive T lymphocytes and initiate adaptive immune responses [8,9]. Macrophages and neutrophils can actively contribute to the elimination of pathogens from the peritoneal cavity with many weapons such as phagocytosis, the production of reactive oxygen species, the formation of extracellular traps, and cytotoxic ability [10,11,12,13,14]. The composition of peritoneal leukocytes in fish has been discriminated according to morphology in previous studies [1,3]. However, the proportion of each cell type cannot be identified because of the lack of antibody tools. In previous studies, we developed antibodies (Abs) against flounder CD4-1, CD4-2 [15], IgM [16], CD83 [8], MHCII [17], and G-CSFR (unpublished) and investigated immune cells in peripheral blood and other immune organs to evaluate the effect of the vaccination [18].

Various studies have observed that antibody-secreting cells increased in the PerC after i.p. administration with bacterium or virus in salmonids [19,20,21]. Intraperitoneal injection seems to be the most effective vaccination route in aquaculture, as many i.p.-administered fish vaccines can confer solid and long-lasting immune responses [19,22]. I.p.-injected oil-adjuvanted commercial vaccines have been successfully used in salmonid farms [23,24]. Compared to other vaccination routes, an i.p. injection vaccine results in rapid changes in the cellular composition in the peritoneal cavity and an increase in the number of neutrophils, macrophages, and other immune cells accompanying robust immune response at both humoral and cellular levels [25,26,27]. Due to the lack of bone marrow in the fish, these rapidly increasing immune cells migrate from the immune organs where immune cells are produced and differentiated to the peritoneal cavity in the action of chemokines [28]. Compared with inactivated bacteria, live bacteria have bacterial virulence factors that can affect the immune response [29]. It is still unknown whether the peritoneal immune response differs in the contexts of infection and vaccination with inactivated pathogens. Thus, further study of the cellular and molecular processes in the challenged peritoneal cavity would enrich the current knowledge of the local immune system.

Flounder (*Paralichthys olivaceus*) is one of the most important aquaculture species in China, Japan, and Korea [30,31]. Commercial injection and immersion vaccines against *Streptococcus iniae* and *Edwardsiella tarda* are available in the flounder farming industry, and i.p. injection vaccination is commonly used [23]. Previous studies have found that the leukocytes in peripheral blood and lymphatic organs such as the head kidney and spleen cause cell activation and differentiation after the vaccine injection [32]. However, there are few studies on the local response in the vaccine injection site and the relationship among local responses in the peritoneal cavity and other immune organs. So, understanding local PerC immune responses in flounder is essential. In this study, we used antibodies against flounder cell surface proteins to analyze PerC leukocytes and characterize the early immune responses in the flounder PerC after i.p. administration with pathogenic *Vibrio anguillarum* and with inactivated bacterial cells.

## 2. Material and Methods

### 2.1. Animals and Bacteria

Healthy flounders (550 ± 100 g) were obtained from a fish farm (Rizhao, Shandong, China) and acclimated in the wet lab with continuously aerated seawater for one week. The animal study was reviewed and approved by the Committee of the Ethics on Animal Care and Experiments at the Ocean University of China. The *V. anguillarum* strain was cultured in 2216E broth for 4 h with shaking at 150 rpm at 28 °C, and then, bacterial cells were collected and washed three times with 0.01 M phosphate-buffered saline (PBS, pH 7.2) via centrifugation at 6000× *g* for 15 min. The *V. anguillarum* with a 1.0 × 10^8^ CFU/mL concentration was used for the challenge experiment. The bacteria suspension with a concentration of 1 × 10^9^ CFU/mL was treated with 0.5% formalin (*v/v*) for 48 h at 30 °C for the inactivation of the bacteria. The inactivated bacteria were washed three times with sterilized PBS via centrifugation at 8000× *g* for 15 min. After the last wash, the concentration of bacteria suspension was adjusted to 1.0 × 10^8^ CFU/mL and stored at 4 °C until use. The effect of inactivation of the bacteria was confirmed by incubating the solution on a 2216E agar plate at 28 °C for 48 h.

### 2.2. Antibodies

Mouse anti-flounder IgM, CD4-1, or CD4-2 monoclonal antibodies (Mabs) [15,16] and rabbit polyclonal antibody against recombinant flounder CD83 [8], MHCII [17], or G-CSFR (unpublished) were stored in our laboratory. The antibodies (1 µg/µL) were diluted into 1:1000 with 1% BSA before they were used in immunofluorescence staining (IFS) and flow cytometry (FCM).

### 2.3. Fish Challenge and Isolation of PerC Cells

The fish were firstly confirmed to be *V. anguillarum*-negative, detected using 16s DNA before the challenge. Forty-five fish were randomly divided into three groups. Control fish (*n* = 15) (designated group C) were injected intraperitoneally with 0.2 mL of 0.01 M PBS. Another fifteen fish were injected with 0.2 mL 1.0 × 10^8^ CFU/mL of living *V. anguillarum* suspension, defined as group V. The remaining fifteen fish were then injected with 0.2 mL of 1.0 × 10^8^ CFU/mL formalin-inactivated *V. anguillarum* suspension, defined as group F. After treatment, three individuals in each group were randomly selected at 0 h, 6 h, 12 h, 24 h, and 48 h post-immunization and infection, respectively. Before the challenge and sampling, the fish were anesthetized with tricaine methanesulfonate (Sigma, St. Louis, MO, USA). To avoid PerC cells from being contaminated by blood cells, blood was removed by drawing it from the caudal vein with a syringe, and then, the fish was injected with 40 mL of PBS in the peritoneal cavity. After gently massaging the abdominal surface, approximately 35 mL of PBS containing PerC cells was harvested with a syringe. After challenge, the peritoneal fluid of fish injected with live bacteria and inactivated bacteria was plate-underlined to check the bacterial presence and infection in recipients of inactivated vaccines to confirm the inactivation of bacteria.

### 2.4. Sorting Cells via Flow Cytometry

Myeloid cells and lymphocytes were separated from total PerC cells via flow cytometry (BD FACSAria, New York, NY, USA) according to FSC and SSC. The purity of harvested cells was analyzed after being sorted (≥98%). The sorted cells were processed for Wright–Giemsa staining, and then the images were taken using a microscope (Olympus, Tokyo, Japan).

### 2.5. Indirect Immunofluorescence and FACS Analysis

After being adjusted to 5 × 10^6^ cells/mL in PBS, the PerCs were blocked with 3% BSA for 1 h at 28 °C and then incubated with primary antibodies (anti-CD4-1, CD4-2, IgM, G-CSFR, MHCII, or CD83) for 1 h at 28 °C, respectively. In parallel experiments, the mouse or rabbit anti-Trx antibodies were used as negative controls. After being washed three times with PBS, the secondary antibodies, goat-anti-mouse Ig-Alexa Fluor^®^ 649 (1:1000, Thermo Fisher Scientific, Waltham, MA, USA), or goat-anti-rabbit Ig-Alexa Fluor^®^ 488 (1:1000, Thermo Fisher Scientific, USA) were applied to incubation for 1 h at 28 °C in the dark. Further staining with DAPI to show nuclei was performed before photomicrography was conducted. Fluorescence images of the samples were taken using a microscope (Olympus DP70, Tokyo, Japan).

### 2.6. Transmission Electron Microscopy

For the ultrastructural analysis, the isolated PerC cells from healthy flounder were centrifugated at 500× *g* for 10 min, and then, the pellets were fixed in 2.5% glutaraldehyde at 4 °C for 2 h. After that, they were post-fixed with 1% (*w*/*v*) osmium tetroxide for 2 h and washed in PBS. The samples were dehydrated in increasing ethanol concentrations and infiltrated with an acetone/resin mixture, and then embedded in resin (Epon 812). The blocks were sectioned, and ultrathin sections (70 nm) were mounted on formvar-coated grids and stained with uranyl acetate and lead citrate for 20 min per step. The cells were analyzed by using TEM (HT-7700, Hitachi, Tokyo, Japan).

### 2.7. PerC Cells Analyzed via Flow Cytometry

The PerC cells were counted and diluted to 5 × 10^6^ cells/mL in PBS. For the detection of IgM^+^, CD4^+^, G-CSFR^+^, MHCII^+^, or CD83^+^ cells, the PerC cells were divided into five parts and incubated with primary anti-CD4-1, CD4-2, IgM, G-CSFR, MHCII, or CD83 antibodies, respectively, and then incubated with goat-anti-mouse Ig-Alexa Fluor^®^ 488 (1:1000, Thermo Fisher Scientific, USA) or goat-anti-rabbit Ig-Alexa Fluor^®^ 488 (1:1000, Thermo Fisher Scientific, USA) for 1 h in the dark at 28 °C, and then washed again. The cell suspensions were then analyzed with an Accuri C6 cytometer (BD, New York, NY, USA). The mouse or rabbit anti-Trx antibodies instead of primary antibodies were used as negative controls.

### 2.8. Quantitative Real-Time PCR

Quantitative real-time PCR (qRT-PCR) was performed using a LightCycler^®^ 480 II Real-Time System (Roche, Basel, Switzerland). Each reaction consisted of 10 μL of 2×ChamQ Universal SYBR qPCR Master Mix (Vazyme, Nanjing, China), 0.4 μL of forward primer and 0.4 μL of reverse primer (10 μM), 2 μL of cDNA, and 7.2 μL of DEPC H_2_O. The qRT-PCR primers and housekeeping gene primers are listed in Table 1. The efficiency of the primers was confirmed to be within 90–110%. The expression levels of the target genes were normalized to those of the combination of the two most stable reference genes (18s and β-actin). The relative expression of each reference gene and target gene was calculated, and the two values were calculated as the geometric mean. The relative expression of the genes was calculated using the 2^−ΔΔCT^ method.

### 2.9. Statistic Analysis

The experiments in this study were repeated more three times, and the data were expressed as the mean ± SD. The *p*-value < 0.05 was considered significant. One-way analysis of variance (ANOVA) and Duncan’s multiple comparisons were performed by using Statistical Product and Service Solution (SPSS) software (Version 20.0; SPSS, IBM, BY, USA).

## 3. Results

### 3.1. Composition and Characteristics of PerC Resident Cells

**Figure 1:**
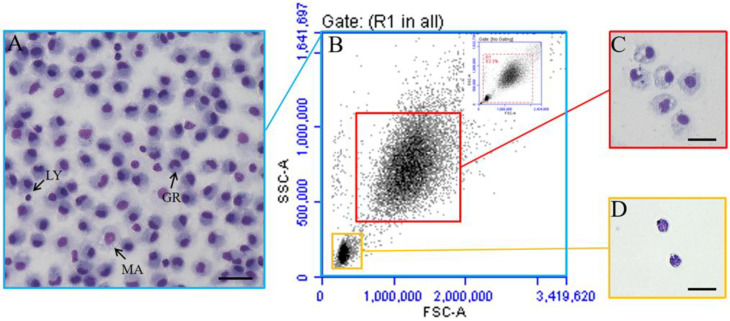
Flow cytometric and Giemsa stain analysis of PerC cells in normal flounder. (**A**) PerC cells were stained with Giemsa. LY: lymphocyte; MA: macrophages; GR: granulocytes. (**B**) Flow cytometric analysis of PerC cells. (**C**) Sorted myeloid cells stained with Giemsa. (**D**) Sorted lymphocytes stained with Giemsa. Bar of both panels: 10 μm.

The flow cytometric analysis of the resident PerC cells revealed two populations that varied in granularity and size (Figure 1). One population of the cells, approximately 15.43% ± 1.8%, was smaller with a lower granularity and designated as lymphocytes (FSC-Hlow and SSC-Hlow). The other population of the cells, about 78.17% ± 3.52%, was larger (FSC-Hhigh) with a higher granularity (SSC-Hhigh) and was designated as myeloid cells (Figure 1B). After sorting via flow cytometry, the two cell populations were successfully separated. The total PerC cells and two separated cell populations were stained with Wright–Giemsa dye. The results showed that the peritoneal cavity in flounder contains different cells, mainly granulocytes, lymphocytes, and macrophages (Figure 1A). The sorted lymphoid cell population possesses a large nucleus with less cytoplasm (Figure 1D). In the other cell population, the nuclei are reniform and dumbbell-shaped with relatively loose cytoplasm (Figure 1C).

**Figure 2:**
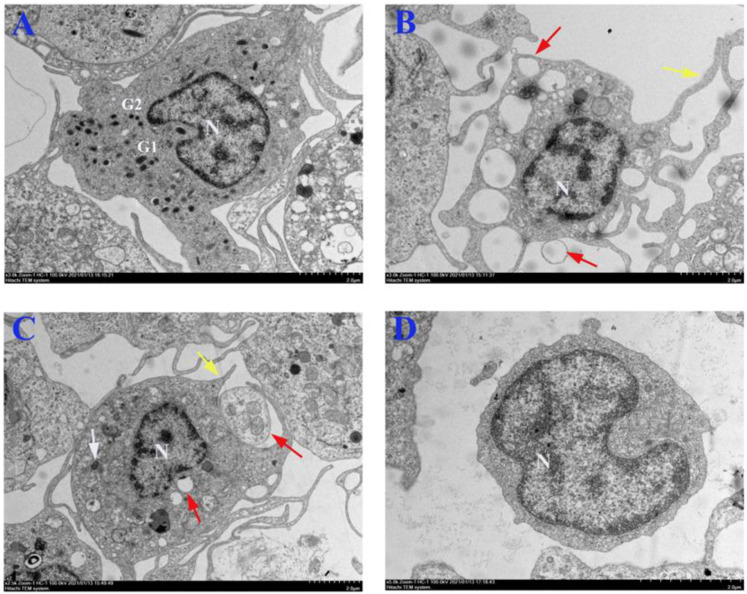
Ultrastructure of the PerC cells in flounder. (**A**) Granulocytes; flounder peritoneal exudate showing first granule-type (GI) and second granule-type (G2) granules; (**B**) dendritic cells; (**C**) macrophages; (**D**) lymphocytes. N represents the nucleus; white arrows indicate lysosomal, yellow arrows indicate pseudopod-like or short protrusions, and red arrows show vesicles inside or released from the cells. Bar of both panels: 2 μm.

Electron microscopy distinguished PerC cells into granulocytes, macrophages, dendritic cells, and lymphocytes based on ultrastructural characteristics (Figure 2). The granulocytes exhibited an eccentric, slightly indented, or bi-lobed nucleus with a small rim of heterochromatin. A notable cytoplasmic feature was the presence of two granule populations differentiated by their electron density. One of the granule populations consisted of oval or round granules containing a homogeneous, granular or fibrous, medium electron-dense material (Figure 2A, G1). In contrast, the other population comprised round granules containing granular, medium electron-dense material [1] (Figure 2A, G2). Dendritic cells possessed a lobed or irregularly outlined nucleus, with dendritic or pseudopod-like protrusions on the cell surface. In addition, dendritic cells showed many vesicles that appeared to release extracellular space [6,8,33] (Figure 2B). The macrophages had round or oval nuclei, short protrusions on the cell surface, lysosomes, and phagocytic vesicles in the cytoplasm [3] (Figure 2C). The lymphocytes of flounder were rounded and possessed a large nucleus with an eccentric nucleolus [3] (Figure 2D).

**Figure 3:**
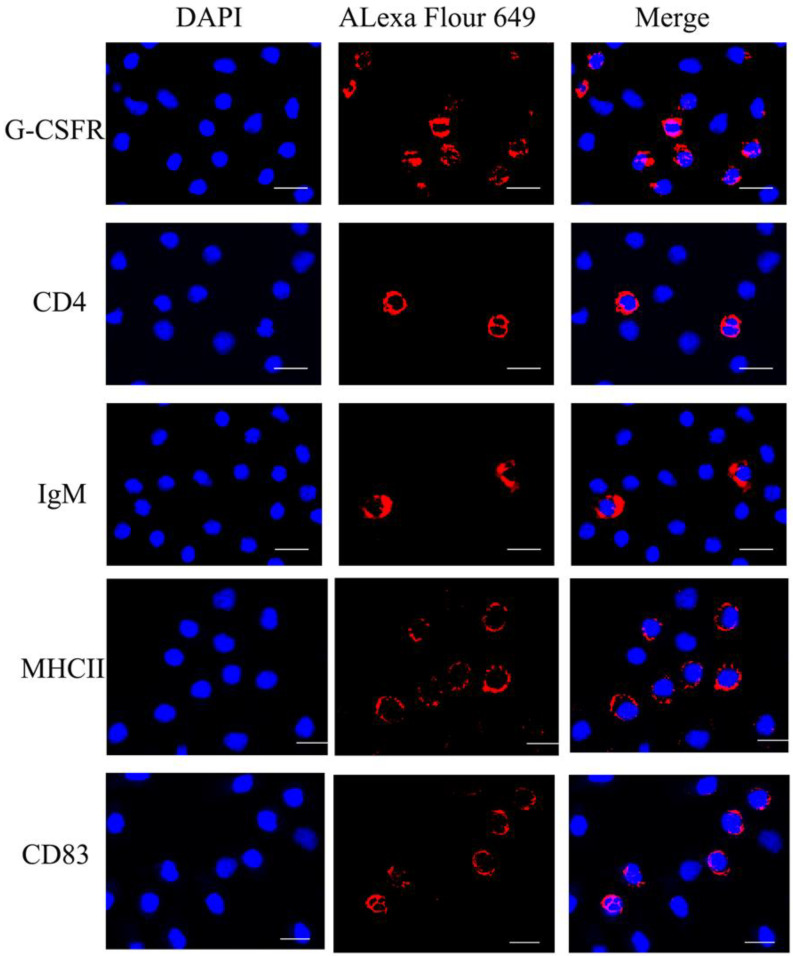
Immunofluorescence staining results for G-CSFR^+^, IgM^+^, CD4^+^, MHCII^+^, and CD83^+^ cells among PerC cells. The red color shows that Alexa Flour 649 labeled antibodies and linked with the cell marker proteins. The nuclei were counterstained in blue with DAPI. Bar of both panels: 10 μm.

**Figure 4:**
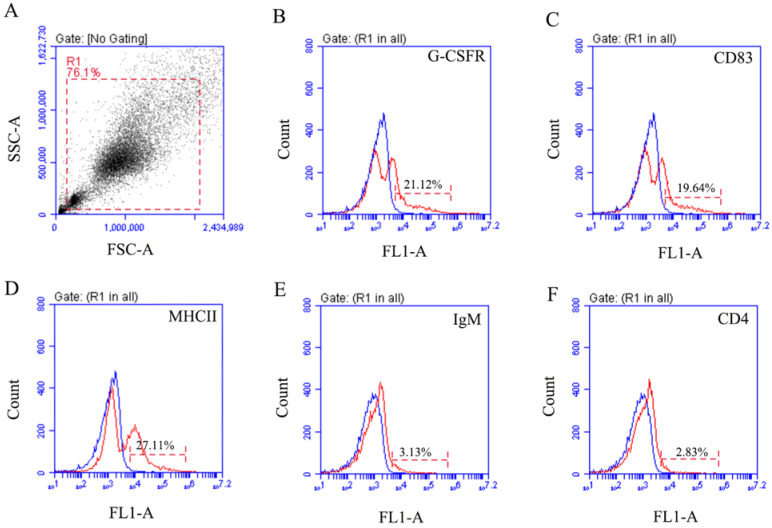
Flow cytometric analysis of the proportions of IgM^+^, CD4^+^, G-CSFR^+^, MHCII^+^, and CD83^+^ cells among PerC cells. (**A**). PerC cells gated on a forward scatter (FSC) versus side scatter (SSC) dot plot; (**B**–**F**): fluorescence histogram of gated peritoneal cells in G-CSFR^+^, MHCII^+^, CD83^+^, IgM^+^, CD4^+^ cells.

Fluorescence microscopy showed that PerC cells have the signals of IgM-, CD4-, G-CSFR-, MHCII-, and CD83-positive leukocytes (Figure 3). The IgM^+^ and CD4^+^-positive cells indicated that the peritoneal cavity exists B and T lymphocytes, respectively. G-CSFR-positive signal could also be detected on PerC cells, indicating that granulocytes, especially neutrophils, existed in the peritoneal cavity. Moreover, MHCII was expressed on dendritic cells, macrophages, and B cells. In addition, CD83 is considered as a cell surface marker of mature dendritic cells in mammals. Flow cytometry analysis reported that the percentages of IgM^+^, CD4^+^, G-CSFR^+^, MHCII^+^, and CD83^+^ leukocytes in peritoneal cells were 3.13% ± 0.4%, 2.83% ± 0.53%, 21.12% ± 1.44%, 27.11% ± 3.30%, and 19.64% ± 0.31%, respectively (Figure 4). These findings suggested that the peritoneal cells in flounder are dominated mainly by granulocytes, macrophages, lymphocytes, and dendritic cells.

### 3.2. Composition Changes in PerC Cells after Infection and Immunization

**Figure 5:**
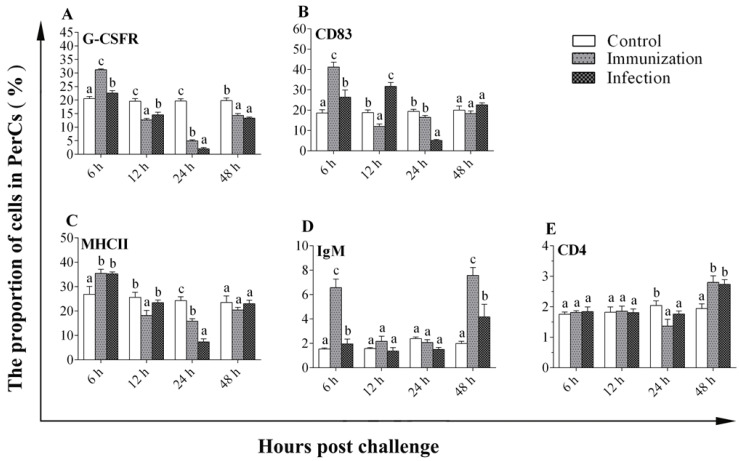
Proportions of different cells in the peritoneal cavity included G-CSFR^+^(**A**), CD83^+^(**B**), MHCII^+^(**C**), IgM^+^ (**D**), and CD4^+^(**E**) cells. Results are expressed as means ± SD of three replicates. Different letters on the bars denote the statistical significance (*p* < 0.05).

To compare the early immune response of PerC cells upon infection and immunization, the proportions of IgM^+^, CD4^+^, G-CSFR^+^, MHCII^+^, and CD83^+^ cells were measured via flow cytometry at different time points after challenge and immunization (Figure 5). The percentages of G-CSFR^+^ and MHCII^+^ peaked at hour 6 (*p* < 0.05), gradually decreased until hour 24 (*p* < 0.05), and then increased at hour 48 post live and formalin-inactivated *V. anguillarum* injection (Figure 5A,C). The percentage of CD83^+^ cells in the immunization group reached the peak at hour 6 (*p* < 0.05), decreased at hour 12 (*p* < 0.05), and then the rate slowly increased to the level of the control group at 48 h. The percentage of CD83^+^ cells in the infection group increased at 12 h (*p* < 0.05) and reached a maximum (*p* < 0.05), followed by decreasing to the lowest point at hour 24 (Figure 5B). The percentage of IgM^+^ cells showed an increase at hour 6 (*p* < 0.05), gradually decreased until hour 24, and then increased to the peak at hour 48 post live and formalin-inactivated *V. anguillarum* injection (Figure 5D). In contrast, after immunization and infection with *V. anguillarum*, the percentage of CD4^+^ cells did not change significantly within the first 12 h (*p* > 0.05), was lightly decreased at hour 24 (*p* < 0.05), and then increased to the peak at 48 h (*p* < 0.05) (Figure 5E). These different response patterns indicate that the different cells may play varied roles in early immune responses in the PerC.

### 3.3. Gene Expression of PerC Cells in Response to V. Anguillarum Infection and Vaccination

**Figure 6:**
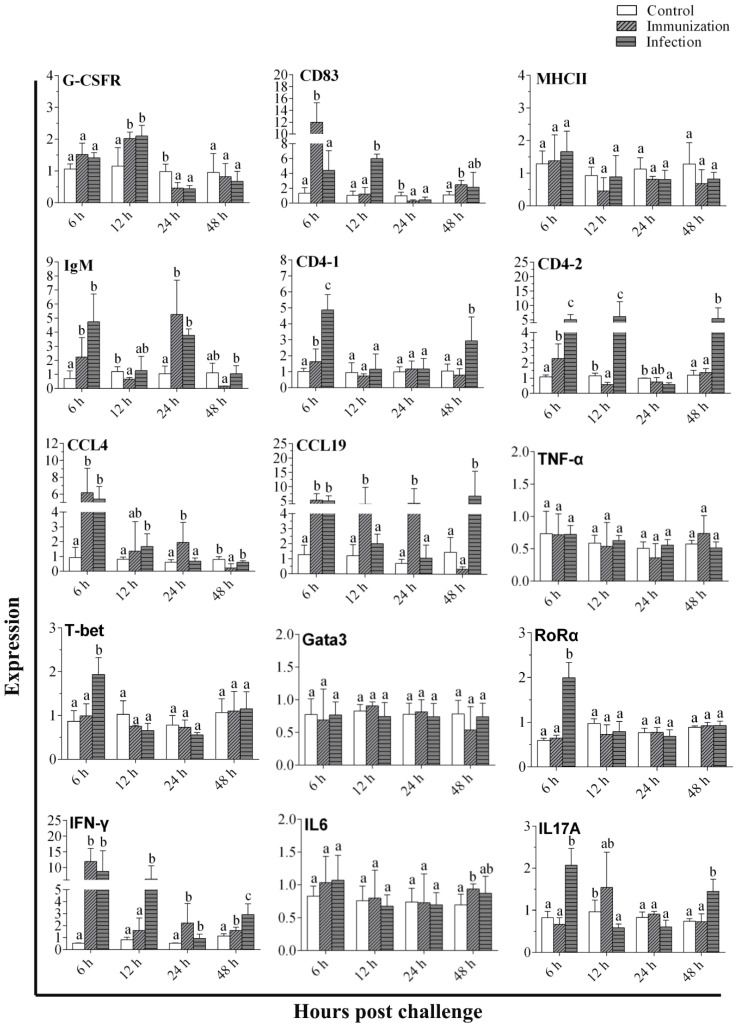
Analysis of the genes in response to *V. anguillarum* infection, vaccination, and PBS in PerC cells. The results are presented as the means ± SD. Different letters on the bar represent the statistical significance (*p* < 0.05) of difference between the experimental and control groups.

The expression levels of selected genes in total PerC cells after *V. anguillarum* infection and vaccination are summarized in Figure 6. The qRT-PCR analysis showed that *V. anguillarum* infection and vaccination induced the up-regulation of membrane molecules, transcription factors, and cytokine genes compared to the control group (*p* < 0.05). The G-CSFR was gradually up-regulated and peaked at 12 h (*p* < 0.05) after infection and immunization, then declined to normal levels, while the expression level was slowly increased to near the level of the control group until hour 48 (*p* > 0.05) (Figure 6). The CD83 gene in the immunization group reached the peak at hour 6 (*p* < 0.05), whereas, in the infection group, it reached a maximum at hour 12 (*p* < 0.05) (Figure 6). Compared to the control groups, there was no significant change in MHCII, TNF-α, IL6, and Gata3 mRNA levels at any sampling time points (*p* > 0.05) (Figure 6). The expression peak times of the IgM genes appeared at 6 h and 24 h post-infection or immunization (*p* < 0.05) (Figure 6). The CD4-1 and CD4-2 genes showed an overall trend of increasing then decreasing after infection and immunization (Figure 6). The CCL19 and CCL4 genes showed a similar trend, and they all peaked at 6 h (*p* < 0.05) and then decreased after infection and immunization (Figure 6). The IFN-γ, T-bet, RORα, and IL17A genes were up-regulated and peaked at 6 h (*p* < 0.05) after infection and immunization, then declined and then rose again at 48 h (Figure 6).

## 4. Discussion

The results of this study showed granulocytes, macrophages, and dendritic cells dominated the peritoneal cavity in flounder, whereas lymphocytes only constituted a tiny fraction. This was similar to the cell composition in sea bream (*Sparus aurata*) and sea bass (*Dicentrarchus labrax*) [1,34]. However, the composition seems species-specific, and myeloid cells (granulocytes, macrophages, and dendritic cells) do not always represent the dominant cell type in fish. For example, in barramundi (*Lates calcarifer*), the number of myeloid cells and lymphocytes in the peritoneal cavity was equal [5], while in rainbow trout, lymphocytes comprised the predominant cell type and myeloid cells only represented a tiny fraction [35,36]. At present, the reason for and consequences of these differences remain unclear.

The ultrastructure of PerC cells showed that the cell morphology and composition of flounder PerC cells are similar to the PerC cells isolated from other fish species [1,3]. Specifically, the granulocytes have a notable feature, the particulate granule, which plays pivotal roles in cellular redox homeostasis and the response to oxidative stresses [37,38,39]. The granulocytes among PerC cells in flounder exhibited two granule types differentiated by their electron density, in line with the granulocytes in sea bream and sea bass [1,3]. The dendritic cells possess many dendrites or pseudopodoid protrusions, which capture antigens and form some complexes, and then are transported to the cell’s surface for recognition by T lymphocytes, thus completing antigen presentation and activating primary T lymphocytes [8,40]. The ultrastructure of dendritic cells shows many vesicle structures that appear to release extracellular vesicles. In line with the investigations in humans, these extracellular vesicles seemed to allow the efficient activation of T lymphocytes, thus displaying potential as promoters of adaptive immune responses [33]. The peritoneal cavity is the primary source of macrophages isolated from mammals [41]. In flounder, macrophages also comprised a large proportion of PerC cells. The primary function could be to enact phagocytosis and digestion on cell debris and pathogens to activate lymphocytes or other immune cells to respond to pathogens [37]. Fish lymphoid cells can be subdivided into lymphoblasts (large lymphocytes), lymphocytes (small lymphocytes), and plasma cells by using morphological criteria [42]. Our results showed that small lymphocytes are the primary type of lymphoid cells found in the PerC cells of flounder, and in terms of ultrastructure, these cells are similar to those present in other teleost species. Only one noticeable difference could be established between lymphocytes in flounder and those in the lymphocytes of seabream and sea bass: the latter had a few small dense cytoplasmic granules [1]. Fish lymphocytes play a central role in humoral immunity. However, the absence of plasma cells in the PerC cells of fish and the low percentage of intraperitoneal lymphocytes suggest that the fish peritoneal cavity may not be involved in antibody production.

A unique set of monoclonal antibodies or polyclonal antibodies has been employed to identify significant leukocyte subpopulations. In our previous study, rabbit anti-CD83 polyclonal antibodies and mouse anti-IgM, CD4-1, and CD4-2 monoclonal antibodies in flounder were produced to label dendritic cell, B lymphocyte, and T lymphocyte subsets, respectively [8,15,16]. CD83 molecule is highly expressed explicitly on the surface of mature dendritic cells [30]. The CD83-positive cells were found in flounder peritoneal cells in this study. Although dendritic cells have been shown to be effective in activating T lymphocytes, research on their properties and function is limited due to their low levels in fish [43,44,45]. In mammals, dendritic cells (DCs) are usually obtained by culturing precursor cells from bone marrow, but teleost lacks bone marrow [46]. Therefore, the dendritic cells in teleosts are usually cultured in vitro from the head kidney and spleen [8]. In our study, CD83-positive cells were found in peritoneal cells, which may provide a good cell source for the study of dendritic cells. GCSF is a crucial regulator and possesses a wide range of neutrophil functions such as migration, antimicrobial activity, and neutrophil survival. GCSF signaling is initiated by the GCSF receptor, which is required to maintain normal neutrophil numbers during basal and emergency granulopoiesis in mice and zebrafish [47]. The MHC molecules are cell-surface proteins associated with self-and non-self-recognition by T-cell-mediated immunity [48]. The expression of MHCII may be observed in cell surfaces of the leukocyte lineage, including dendritic cells, macrophages, and B cells, where its primary function of presenting antigenic peptides to CD4^+^ T cells has been determined [49,50]. In this study, immunofluorescence staining and FCM studies were performed to confirm that IgM^+^, CD4^+^, CD83^+^, G-CSFR^+^, and MHCII^+^ cells are present in peritoneal cells. Thus, these positive molecules demonstrate the presence of B lymphocytes, T lymphocytes, dendritic cells, granulocytes, and macrophages in the peritoneal cavity. Furthermore, the number of dendritic cells, granulocytes, and macrophages was more prevalent than lymphocytes in healthy flounder, which proved that the peritoneal cavity is an essential site for phagocytic antigens and antigen presentation.

After i.p. immunization and infection, the immune response of PerC cells in the peritoneal cavity showed a similar trend, including two complete changes in the dominating leukocyte populations early within 48 h. The first changes were caused by the rapid recruitment of neutrophils, dendritic cells, and macrophages within the first 6 h, and then, the number of these phagocytes gradually decreased until 24 h, followed by increasing to normal levels by 48 h. Meanwhile, the number of lymphocytes increased at 48 h. Salmonid fishes also have observed increased numbers of antibody-secreting cells which increased in the PerC after i.p. challenge with bacterium or virus [19,20,21]. For the first 6 h after the challenge, G-CSFR^+^ cells representing neutrophils were rapidly chemotaxied to the stimulation site, and then, the number of neutrophils was decreased at 12 and 24 h. It is speculated that the neutrophils would form pus cells and die after the pathogen is engulfed [51]. The variations in CD83^+^ and MHCII^+^ cells representing dendritic cells and macrophages were similar to the change in G-CSFR^+^ cells. At 6 h after the challenge, the number of CD83^+^ and MHCII^+^ cells also increased rapidly; the antigen was engulfed and transported to the surface of the cell to activate primary T lymphocytes [9,37]. The number of CD83^+^ and MHCII^+^ cells dropped during 12 and 24 h, indicating that MHCII molecules with antigens and dendritic cells homed to stimulate T lymphocyte cells [9,48].

*V. anguillarum* is a common pathogenic bacterium in the aquaculture industry and can secrete extracellular products, such as exotoxins, to interfere with or evade local phagocytosis to establish infection [52]. In this study, inactive *V. anguillarum* induced more G-CSFR^+^, CD83^+^, and IgM^+^ cells in the peritoneal cavity than live *V. anguillarum* at 6 h after the challenge. Although there were changes at other time points, overall, the infection of live *V. anguillarum* did not increase the proportion of immune cells detected in this study compared to the inactive *V. anguillarum* immunization. It can be conjectured that live *V. anguillarum* can grow and proliferate in fish, camouflage antigens to make *V. anguillarum* elude phagocyte phagocytosis, or release exotoxins to reduce or delay the chemotaxis in the peritoneal cavity. All levels of G-CSFR, CD83, and MHCII recovered to normal by 48 h, suggesting that they might ease the non-specific immune stage. The IgM and CD4 levels were elevated at this time point, indicating that the signal transduction of a specific immune response was increased.

Compared to the results for the positive cells measured via flow cytometry among peritoneal cells after *V. anguillarum* infection and immunization, the qRT-PCR results showed a similar changing trend at the gene level. In addition to analyzing the changes in the membrane molecular genes, the gene expression of several cytokines and transcription factors, CCL19, CCL4, TNF- α, IFN- γ, IL6, T-bet, RORα, IL17A, and Gata3, were also determined. Chemokines CCL4 and CCL19 participate in the local inflammatory reaction and play a chemotaxis role and can taxis a variety of immune cells such as neutrophils, monocytes/macrophages, dendritic cells, and natural killer cells (NKs) and migrate to the site where inflammation occurs [32]. CCL19 and CCL4 both peaked at 6 h and then declined. Consistent with the flow results, the leukocytes were rapidly chemotactic to the site of inflammation after infection and immunization. IFN- γ, IL17A, the cytokine secreted by activated T cells [53,54], shows a trend of rising at 48 h, which means that activating T cells at 48 h strengthens specific immunity. T-bet, Gata-3, and RORα were shown to be the transcription factors, specifically targeting T cells, and could make T cells differentiate into helper T cell type 1, helper T cell type 2, and helper T cell type 17, respectively [55,56,57,58]. The expression of T-bet, RORαalso increased at 48 h, which is also proved to enter a specific immune stage. IL-6 corresponds to the function of B cells, and TNF- α is a pro-inflammatory cytokine, which participate in both inflammatory and immune responses [59,60]. Both genes showed no significant change. Together, these results suggested that the early activation of peritoneal cells in response to *V. anguillarum* infection and vaccination are divided into two stages: a non-specific stage dominated by phagocytes and a specific immune stage dominated by lymphocytes. Moreover, due to its virulence, the live *V. anguillarum* causes a more intense and complicated PerC cells immune response than inactive *V. anguillarum*.

## 5. Conclusions

In summary, cells are dominated by granulocytes, macrophages, and dendritic cells in the peritoneal cavity of normal flounder. The early activation of PerC cells includes two stages, a non-specific stage dominated by phagocytes and a specific immune stage dominated by lymphocytes in response to *V. anguillarum* infection and vaccination in fish. Live *V. anguillarum* with virulence factors causes a more intense and complex PerC cell immune response than the inactive *V. anguillarum*. Together, this is the first study on the morphology and composition of PerC cells and early peritoneal cavity immune response in flounder to *V. anguillarum* infection and immunization with killed bacterium. This study advances the understanding of the cellular dynamics of local immune responses in the peritoneal cavity which will inform future vaccination strategies. 

## Figures and Tables

**Table 1:** Primers used in the present study.

Gene	Primer Sequence (5′-3′)	Accession Number
*18sRNA*	F: GGTCTGTGATGCCCTTAGATGTC R: AGTGGGGTTCAGCGGGTTAC	EF126037
*β-actin*	F: GAGGGAAATCGTTCGTGACAT R: ATTGCCGATGGTGATGACCTG	AF135499.1
*CD4-1*	F: CCAGTGGTCCCCACCTAAAA R: CACTTCTGGGACGGTGAGATG	AB643634
*CD4-2*	F: CACAGCGAGGACGTCAGAAA R: TCTCTCCCATCACTCCTTTAGCA	AB640684
*IgM*	F: AAGTCCACAAATTACCCTCCAA R: TTCTCGCTTTTATGTTCCTCAG	AB052744
*CD83*	F: CCCAACGGCACGACGACATAC R: CCCAAAGGTGCTGCCAGGTGA	KR998303.1
*G-CSFR*	F: ACCTCCCCACCCAGTACACC R: AGTTCATTCACCGCCTTCACAT	109626803
*MHCII*	F: GACGGTGAAGAGATGTGGTT R: ATCGGACTGGAGGGAGGC	AY99753
*CCL19*	F: GACATCAGCACAGGTTCCCA R: GGATGGTGGCGTCGATAGAG	AB937788.1
*CCL4*	F: ATGCTGGCTGCCATTACTGT R: CATGTAGCCGACCACCTTGT	AB937786.1
*TNF-α*	F: TCCTGGCGTTTTCTTGGT R: TGGCTCTGCTGCTGATTT	AB040448.1
*IL6*	F: CAAAGGTTGGCTGAAGGC R: TGGAAAGTGCTGGGGTTG	DQ267937.1
*IFN-γ*	F: TGGTCTGTCTGTCCCTGTG R: GCTTCCCGTTGAATCTGT	AB435093.1
*Gata3*	F: CAGGAGGACAAAGAGTGCATAAAGT R: GAAGATGACCCACCTATCAGGCTAC	XM_020108979.1
*T-bet*	F: GCCGACATCAGCAGTCACCT R: TGTGCGTAAAACCTGCCG	KR822591.1
*RORα*	F: CCTTACTGCTCCTTCACCAACG R: GGCGAACTCCACCACATACTG	XM_020079419.1
*IL17A*	F: CCTGGATGTGACTCCTTGTTGG R: GACGCTCTGGTAGATGGGAACT	XM_020111881.1

## Data Availability

The data in this study are readily available upon reasonable request to the corresponding author.
